# Preoperative waiting time and no-show risk in day surgery: a large-scale cohort study

**DOI:** 10.3389/fmed.2026.1810536

**Published:** 2026-04-20

**Authors:** Xiaoyan Wu, Qianyin Zhu, Meijuan Lan, Leiwen Tang, Huadi Yuan, Xiajuan Jiang, Li Liu, Dingjie Xin

**Affiliations:** 1 Department of Nursing, The Second Affiliated Hospital of Zhejiang University School of Medicine, Hangzhou, Zhejiang, China; 2 Department of Artificial Intelligence and Information Technology, The Second Affiliated Hospital of Zhejiang University School of Medicine, Hangzhou, Zhejiang, China

**Keywords:** day surgery, no-show risk, patient attendance, preoperative waiting time, surgical scheduling

## Abstract

**Background:**

No-shows in day surgery represent a global challenge that undermines healthcare efficiency, leading to substantial resource waste and increased operational costs. Preoperative waiting time is a key factor influencing patient attendance. This study investigated the association between preoperative waiting time and the risk of day-surgery no-shows.

**Methods:**

This retrospective cohort study included 79,516 day-surgery patients. Demographic characteristics, surgical information, and preoperative waiting time were extracted from the electronic medical record system. Patients were categorized into quartiles according to waiting time: Q1 (0–<3 days), Q2 (3–<6 days), Q3 (6–<11 days), and Q4 (≥11 days). Multivariable logistic regression and restricted cubic spline (RCS) analyses were performed to quantify the association between preoperative waiting time and no-show risk.

**Results:**

Among the 79,516 patients undergoing day surgery, 2,009 (2.53%) experienced no-show. The median waiting time was 6 (3–11) days. After adjusting for confounding factors, longer preoperative waiting was significantly associated with increased odds of no-shows(*p* < 0.001). RCS analysis revealed a nonlinear dose–response relationship (*p* for trend <0.001; *p* for nonlinearity <0.001). The no-show risk rose sharply when waiting time exceeded approximately 6 days.

**Conclusion:**

Our data showed a significant association between longer preoperative waiting time and an increased risk of no-show among day-surgery patients. This association became more apparent when the preoperative waiting time exceeded approximately 6 days. These findings provide preliminary quantitative evidence regarding the relationship between preoperative waiting time and no-show risk in day surgery.

## Introduction

Surgical no-show refers to a patient’s failure to receive scheduled medical services on the planned day of surgery without prior cancellation. Reported no-show rates vary substantially across countries and hospital types, ranging from 6.6 to 28% (1). In most healthcare systems worldwide, surgical no-shows have become a persistent burden, significantly constraining revenue growth, increasing operational costs, and causing substantial waste of medical resources. This issue poses a major global health challenge (2, 3).

In China, strained medical resources and challenges in access to care underscore the importance of efficient models (4). Day surgery, defined as the process in which admission, surgery, and discharge are completed within 24 h (5), has been regarded as an optimal strategy to improve resource allocation. It effectively shortens length of stay, reduces medical costs, and enhances bed turnover efficiency (6). However, the occurrence of surgical no-shows undermines these potential benefits.

No-show behavior is a complex outcome shaped by multiple factors, which can be broadly categorized into patient-related factors (e.g., forgetting appointments or scheduling conflicts) and hospital-related factors (e.g., long waiting times, insufficient reminders, appointment management issues, and patient–provider relationship challenges) (7, 8). Studies show that more than 80% of surgical cancellations are potentially avoidable, with only about 20% attributed to uncontrollable causes (9). Among modifiable risk factors, preoperative waiting time, the interval from scheduling to the actual surgical date, has been associated with no-show behavior (3, 10). Werner et al. (11) demonstrated that prolonged waiting time may diminish patients’ belief in the benefits of surgery, reduce perceived behavioral advantages, and amplify perceived barriers (e.g., time cost, discomfort), thereby increasing no-show risk. Longer waits may also lead to forgotten appointments, scheduling conflicts, or seeking care elsewhere (7, 10).

However, existing research has primarily remained descriptive. Although prior studies have confirmed an association between waiting time and no-show behavior, the dose–response relationship and specific risk thresholds remain unclear. It is not yet known how different durations of waiting time quantitatively influence no-show risk among day surgery patients, nor have targeted nursing interventions been established to mitigate the adverse effects of extended waiting time. Furthermore, no standardized guideline exists for managing preoperative waiting time in day surgery.

Therefore, this study aimed to use large-scale real-world data and construct a restricted cubic spline (RCS) model to quantify the relationship between preoperative waiting time and no-show risk, identify potential inflection points in risk, and inform future nursing and management strategies. The goal is to provide empirical evidence and precise intervention pathways to optimize day surgery workflows and reduce no-show rates.

## Materials and methods

### Study design

This study was a retrospective cohort analysis based on anonymized electronic medical records. The study design and reporting followed the guidelines of the Strengthening the Reporting of Observational Studies in Epidemiology (STROBE) statement (12). The study population was derived from the day surgery management system of a tertiary general hospital in Zhejiang Province, which performed 49,000 day surgeries in 2023. It is one of the most efficient public hospitals in China and is representative in terms of day surgery volume and operational model. Consecutive cases of day surgery from January 2023 to June 2024 were included to investigate the association between preoperative waiting time and the risk of no-shows among day surgery patients. In this study, a no-show was defined as failure to receive scheduled medical services on the planned surgical date without prior cancellation. Cancellations caused by hospital-related factors were not included. Hospital-related reasons specifically included temporary operating room malfunction, medical equipment problems, and situations in which the primary surgeon was unable to perform the surgery as scheduled because of emergency surgery or other unexpected events. In this study, preoperative waiting time itself was treated as the exposure variable of interest rather than being directly classified as a hospital-related reason for surgical cancellation, and was analyzed to explore its association with the risk of no-show.

### Study population

Patients were included if they met the following criteria: (1) They satisfied the admission criteria of the hospital’s *Day Surgery Management Protocol*, including age between 14 and 75 years, being conscious, no history of psychiatric disorders, and no severe underlying disease; (2) Their American Society of Anesthesiologists (ASA) physical status classification was lower than class III, and they met the eligibility criteria of the National Recommended Catalogue for Day Surgery (2022 edition) (13); (3) Their clinical data were complete. Exclusion criteria were: (1) Patients who canceled or rescheduled their surgery within 24 h before the procedure through a phone call. (2) Outpatient procedures, such as superficial skin mass excision, debridement and excision of necrotic skin and subcutaneous tissue, and medial canthoplasty. (3) Interventional procedures, such as transcatheter embolization for arteriovenous malformation of the right forearm, radiofrequency ablation of the great saphenous vein, and prostate brachytherapy seed implantation. (4) Diagnostic or therapeutic procedures, such as transurethral ureteral stent placement, cystoscopy, and hysteroscopy combined with diagnostic curettage. (5) Patients with severe comorbidities such as myocardial infarction, heart failure, arrhythmia, coronary heart disease, coronary stent placement, acute upper respiratory infection, or acute asthma. Because such conditions fall outside the routine admission criteria for standardized day surgery and could introduce substantial clinical heterogeneity related to perioperative instability. (6) To reduce heterogeneity in the cohort, patients whose surgery was canceled for hospital-related reasons were excluded.

### Data collection

The hospital’s day-surgery scheduling process generally followed these steps. After being evaluated by the outpatient clinician and confirmed to meet the indications for day surgery, the patient was issued a surgical appointment form. The patient or a family member then completed registration at the day surgery appointment center. In principle, surgical dates were assigned on a first-come, first-served basis, while also being coordinated according to the operating schedule of the primary surgeon, operating room availability, and the patient’s personal time preferences.

Demographic, surgical, and system-level characteristics were collected. Demographic information included gender, age, health insurance type, current address, education level, and number of children. System information included preoperative waiting time. Surgical information included grade of surgery and anesthetic technique. In China, surgical procedures are classified into four grades according to their complexity and risk (14). Grade I surgery refers to low-risk, simple procedures with low technical difficulty, such as hemorrhoidectomy, excision of a tendon sheath cyst of the hand, and minimally invasive rotary excision of breast lesions. Grade II surgery refers to procedures with moderate risk, complexity, and technical difficulty, such as high ligation and stripping of the great saphenous vein, excision of hydrocele of the testis, and unilateral inguinal hernia repair. Grade III surgery refers to relatively high-risk and more complex procedures requiring greater technical expertise and resource utilization, such as laparoscopic cholecystectomy, laparoscopic repair of indirect inguinal hernia, and endoscopic lumbar discectomy, as well as procedures involving grafts or prostheses. Grade IV surgery refers to highly complex, high-risk procedures requiring substantial resources or involving major ethical considerations, such as thoracoscopic lobectomy, total hip arthroplasty, and total knee arthroplasty. In this study, the surgical grade was automatically assigned by the hospital surgical management system according to standardized coding rules.

Disease diagnoses and surgical procedures were coded according to the International Classification of Diseases, Tenth Revision (ICD-10), and the International Classification of Diseases, Ninth Revision, Clinical Modification for Operations and Procedures (ICD-9-CM-3).

The primary outcome of this study was the occurrence of a no-show on the scheduled surgical date. To avoid correlation among repeated observations, only the first day surgery and the first no-show record during the study period were included for patients who underwent multiple day surgeries. All data were extracted from the hospital’s day surgery management system and surgical scheduling system, and were stored in Microsoft Access. Stata MP version 15.1 was used to link datasets with anonymized identifiers and perform statistical analyses.

### Quality control

Demographic data, surgery-related information, and no-show records were obtained from the day surgery management system and the surgical scheduling system. During data collection, all variables were checked for completeness, and cases with more than 20 percent missing data were removed to ensure the reliability of the final analytical dataset.

During data entry, a strict quality control process was implemented. Predetermined value ranges and logical validation rules were built into the database to prevent entry errors. A double data entry workflow was used, in which two staff members independently entered the same dataset. Consistency was verified through system comparison. Records with discrepancies or questionable information were reviewed manually by checking the original medical documents to ensure accuracy and logical consistency. To reduce the influence of potential bias on the study results, several measures were taken. First, to minimize selection bias, all eligible day-surgery patients during the study period were consecutively included. Second, to minimize information bias, all variables were extracted from standardized electronic medical records, and no-show status was automatically recorded by the hospital information system. Third, to reduce confounding bias, potential demographic, socioeconomic, and clinical confounders were adjusted stepwise in the multivariable regression models, and subgroup analyses were performed.

### Ethics

This study was reviewed and approved by the Ethics Committee of the Second Affiliated Hospital of Zhejiang University School of Medicine (Approval No. 20251426). All data were fully anonymized before analysis. Data processing and analysis were conducted on secure, encrypted computers to ensure information safety. Given the retrospective nature of the study and the de-identified dataset that did not allow individual identification, the requirement for informed consent was waived.

### Statistical analysis

The study population was stratified into quartiles according to preoperative waiting time: Q1 (0 to <3 days), Q2 (3 to <6 days), Q3 (6 to <11 days), and Q4 (≥11 days). Normally distributed continuous variables were expressed as mean ± standard deviation, and comparisons among groups were performed using one-way analysis of variance (ANOVA). Non-normally distributed continuous variables were presented as median (interquartile range), and group comparisons were performed using the Kruskal-Wallis test. Categorical variables were expressed as frequencies and percentages, and were compared using the chi-square test.

Variables considered clinically relevant or found to be statistically significant in the univariate analyses were subsequently entered into the multivariable model. Three logistic regression models were constructed to examine the association between preoperative waiting time and no-show risk. Model 1 was unadjusted. Model 2 adjusted for demographic and socioeconomic variables, including age, gender, health insurance type, current address, education level, and number of children. Model 3 additionally adjusted for grade of surgery and anesthetic technique.

In addition, restricted cubic spline (RCS) analysis was performed as a complementary continuous analysis to flexibly examine the dose–response relationship and potential nonlinearity between preoperative waiting time and no-show risk. Four knots were placed at the 5th, 35th, 65th, and 95th percentiles of preoperative waiting time, corresponding to 1, 4, 8, and 21 days, respectively. Subgroup analyses were performed according to age, gender, health insurance type, current address, education level, number of children, grade of surgery, and anesthetic technique. Statistical analyses were performed using SAS version 9.4, and a two-sided alpha level of 0.05 was considered statistically significant.

## Results

### Baseline characteristics of the study population

A total of 79,516 day surgery patients were included in the final analysis. The overall no-show rate was 2.53%. The median preoperative waiting time of the study population was 6 days (IQR 3–11). The median waiting time in the no-show group was 6 days (IQR 4–13), which was significantly longer than that in the non–no-show group 6 days (IQR 3–11) (*p* < 0.001). The study population was divided into four quartiles: Q1 (0 to <3 days), Q2 (3 to <6 days), Q3 (6 to <11 days), and Q4 (≥11 days). Baseline characteristics differed significantly across preoperative waiting-time quartiles (Table 1). Notably, the no-show rate increased progressively from 1.73% in Q1 to 3.09% in Q4.

**Table 1:** Baseline characteristics of the day surgery patients by preoperative waiting time index quartile.

Variables, *N* (%)	Total (*N* = 79,516)	Q1 (*N* = 14,026)	Q2 (*N* = 25,122)	Q3 (*N* = 19,259)	Q4 (*N* = 21,109)	statistical value	*p-*value
Gender						269.68	< 0.001
Male	29,220 (36.75)	5,357 (38.19)	8,669 (61.81)	6,918 (35.92)	7,848 (37.18)		
Female	50,296 (63.25)	8,669 (61.81)	16,025 (63.79)	12,341 (64.08)	13,261 (62.82)		
Age						924.70	< 0.001
< 45	36,962 (46.48)	6,240 (44.49)	10,439 (41.55)	8,597 (44.64)	11,686 (55.36)		
45–65	33,062 (41.58)	6,205 (44.24)	11,318 (45.05)	8,120 (42.16)	7,419 (35.15)		
≥ 65	9,492 (11.94)	1,581 (11.27)	3,365 (13.39)	2,542 (13.2)	2004 (9.49)		
Health insurance						202.21	< 0.001
Self-pay	8,761 (11.02)	1,323 (9.43)	2,586 (10.29)	1983 (10.3)	2,869 (13.59)		
Medical insurance	70,755 (88.98)	12,703 (90.57)	22,536 (89.71)	17,276 (89.7)	18,240 (86.41)		
Current address						249.93	< 0.001
Non-local	30,856 (38.8)	6,016 (42.89)	10,119 (40.28)	7,314 (37.98)	7,407 (35.09)		
Local	48,660 (61.2)	8,010 (57.11)	15,003 (59.72)	11,945 (62.02)	13,702 (64.91)		
Education level						868.08	< 0.001
High school or below	46,869 (58.96)	8,670 (61.82)	15,878 (63.21)	11,651 (60.51)	10,670 (50.57)		
Associate degree or above	32,630 (41.04)	5,354 (38.18)	9,241 (36.79)	7,605 (39.49)	10,430 (49.43)		
Number of children						576.92	< 0.001
0	17,168 (21.59)	2,698 (19.24)	4,623 (18.4)	3,929 (20.4)	5,918 (28.05)		
1	34,481 (43.37)	6,160 (43.92)	11,284 (44.92)	8,344 (43.33)	8,693 (41.2)		
≥ 2	27,851 (35.03)	5,166 (36.84)	9,212 (36.67)	6,983 (36.26)	6,490 (30.76)		
Grade of surgery						155.11	< 0.001
I & II	35,295 (44.39)	6,785 (48.37)	10,706 (42.62)	8,214 (42.65)	9,590 (45.43)		
III & IV	44,221 (55.61)	7,241 (51.63)	14,416 (57.38)	11,045 (57.35)	11,519 (54.57)		
Anesthetic technique						280.23	< 0.001
Local anesthesia	16,800 (21.13)	3,570 (25.45)	5,271 (20.98)	4,150 (21.55)	3,809 (18.04)		
General anesthesia	62,716 (78.87)	10,456 (74.55)	19,851 (79.02)	15,109 (78.45)	17,300 (81.96)		
No-show						98.12	< 0.001
Yes	2009 (2.53)	242 (1.73)	535 (2.13)	580 (3.01)	652 (3.09)		
No	77,507 (97.47)	13,784 (98.27)	24,587 (97.87)	18,679 (96.99)	20,457 (96.91)		

### Association between preoperative waiting time and no-show risk

As shown in Table 2, longer preoperative waiting time was consistently associated with higher odds of no-show. In the fully adjusted model, compared with Q1, the odds of no-show were higher in Q2 (OR = 1.34, 95% CI 1.15–1.57), Q3 (OR = 2.00, 95% CI 1.71–2.34), and Q4 (OR = 1.98, 95% CI 1.70–2.31). These findings indicate that the positive association remained robust after adjustment for demographic, socioeconomic, and surgical variables.

**Table 2:** Association of preoperative waiting time with no-show risk in day surgery patients.

Variables	Model 1*		Model 2**		Model 3***	
	OR (95% CI)	*p-*value	OR (95% CI)	*p*-value	OR (95% CI)	*p-*value
Preoperative waiting time group		< 0.001		< 0.001		< 0.001
Q1 (0–<3 days)	Ref.		Ref.		Ref.	
Q2 (3–<6 days)	1.24 (1.06–1.45)		1.24 (1.06–1.44)		1.34 (1.15–1.57)	
Q3 (6–<11 days)	1.77 (1.52–2.06)		1.76 (1.52–2.05)		2.00 (1.71–2.34)	
Q4 (≥11 days)	1.82 (1.56–2.11)		1.74 (1.50–2.02)		1.98 (1.70–2.31)	

*Model 1 was unadjusted. **Model 2 was adjusted for age, sex, health insurance, current address, education level, number of children. ***Model 3 was adjusted for age, sex, health insurance, current address, education level, number of children, grade of surgery, anesthetic technique.

### Dose–response relationship between preoperative waiting time and no-show risk

**Figure 1:**
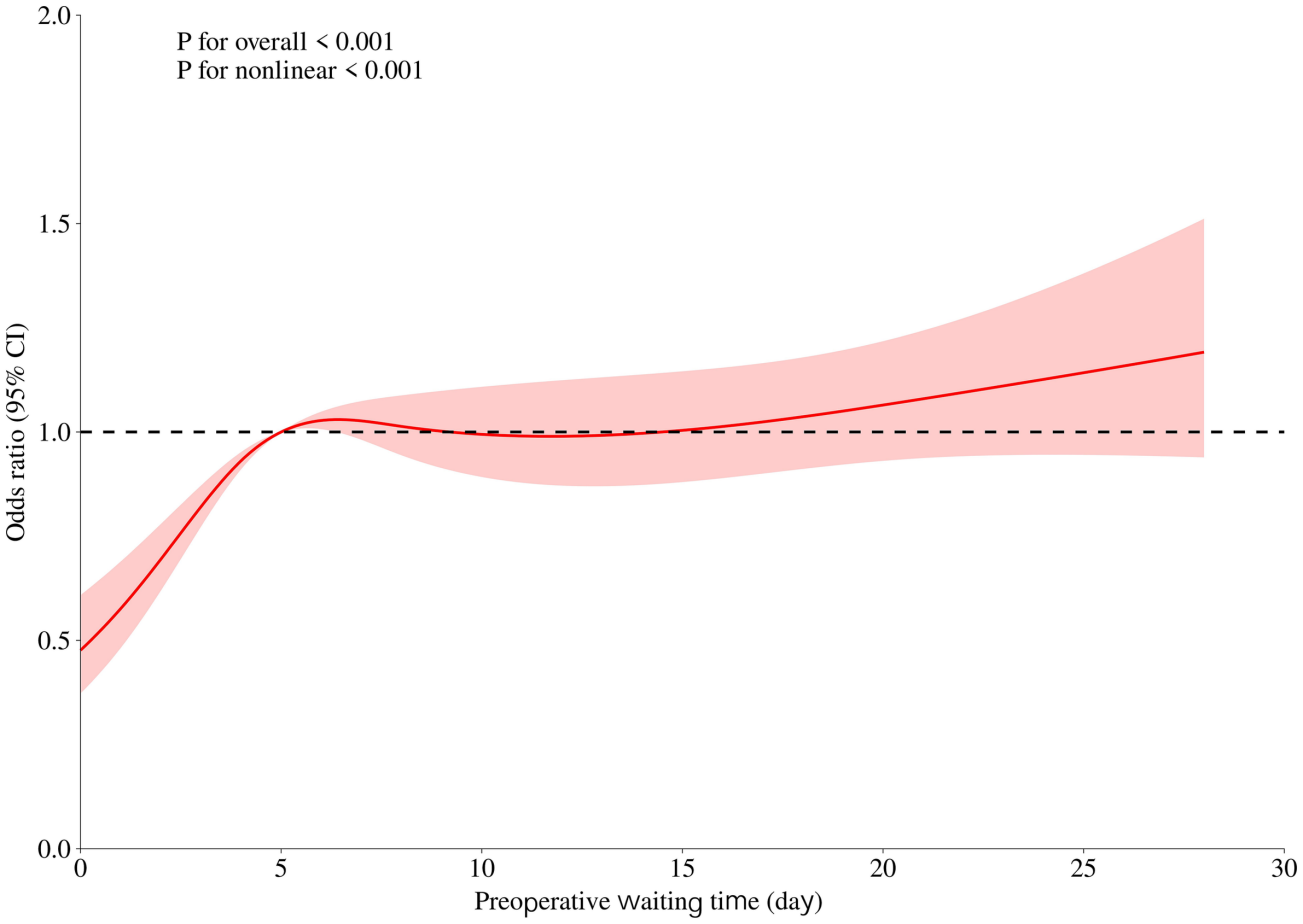
Nonlinear association between preoperative waiting time and no-show risk in the overall study population. Curves represent adjusted odds ratios (ORs) with 95% confidence intervals (shaded areas) derived from multivariable logistic regression models with restricted cubic splines. Four knots were placed at the 5th, 35th, 65th, and 95th percentiles of preoperative waiting time (1, 4, 8, and 21 days, respectively). Models were adjusted for age, gender, health insurance type, current address, education level, number of children, grade of surgery, and anesthetic technique. The dashed horizontal line indicates OR = 1.

The restricted cubic spline analysis in the overall study population revealed a nonlinear association between preoperative waiting time and no-show risk. There was a significant overall association (*p* < 0.001) and significant nonlinearity (*p* < 0.001). When the waiting time exceeded approximately 6 days, the increase in no-show risk became more apparent (Figure 1).

### Subgroup analysis

**Figure 2:**
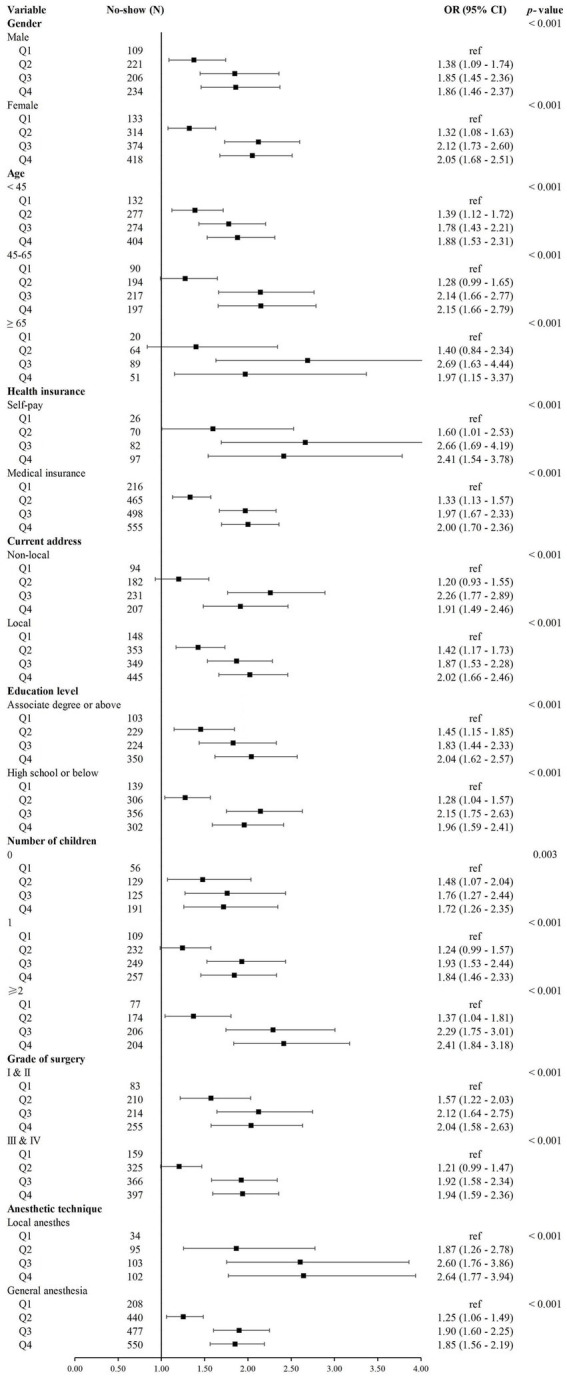
Subgroup analyses of the association between preoperative waiting time and no-show risk in day surgery. Odds ratios (ORs) with 95% confidence intervals (CIs) are presented for quartiles of preoperative waiting time: Q1 (0–<3 days), Q2 (3–<6 days), Q3 (6–<11 days), and Q4 (≥11 days). Estimates were derived from multivariable logistic regression models.

The results of the subgroup analysis revealed that the positive association between preoperative waiting time and no-show risk was generally consistent across subgroups, although the magnitude of the association varied (Figure 2). Relatively stronger associations were observed among patients aged 45–65 years, self-pay patients, those with two or more children, and those undergoing local anesthesia (*p* < 0.001).

## Discussion

This study is the first large-scale investigation to systematically quantify the association between preoperative waiting time and the risk of no-shows in day surgery. The findings demonstrate that waiting time was significantly associated with patient attendance and that longer waiting periods are associated with a progressively higher no-show risk. Multivariable logistic regression revealed a stable and significant increase in risk across quartiles of waiting time, and the restricted cubic spline model further identified a nonlinear dose–response pattern. After adjusting for demographic, socioeconomic, and clinical factors, this association remained robust. These results indicate that preoperative waiting time may be a useful marker in understanding no-show risk.

Preoperative waiting time is a crucial factor influencing healthcare efficiency, patient experience, and clinical outcomes (15–19). It is also a key dimension in evaluating hospital service quality (20). With the expanding scale of day surgery and growing pressure on medical resources in China (4), effective management of waiting time has become essential. The present study extends existing literature by demonstrating a nonlinear relationship and identifying a threshold of approximately 6 days, below which the risk remains low and may even be protective. This provides empirical support for viewing preoperative waiting time may represent a potentially modifiable operational factor.

Unlike previous research focusing on overall association (3, 10), this study reveals risk heterogeneity through subgroup analyses. The elevated risk observed among patients aged 45 to 65 years in the longest waiting group may reflect conflicts between professional responsibilities, family obligations, and scheduled surgery. In contrast, older adults demonstrated peak risk at moderate waiting durations, possibly due to higher dependency on medical care and the tendency to reassess surgical necessity over time. These differentiated patterns align with findings from prior studies, which reported that younger adults benefit more from telemedicine-based reminders and that virtual follow-up can be as effective as in-person visits (21). These insights suggest that no-show reduction strategies should be tailored to specific patient groups.

The mechanisms underlying the association between preoperative waiting time and no-show behavior may be multifactorial. Psychological distress during prolonged waiting, including heightened anxiety and uncertainty, may reduce patient satisfaction and lead some patients to reconsider surgery (4, 22). Socioeconomic constraints, such as work and caregiving burdens, may also intensify over time and contribute to nonattendance (23). From a clinical perspective, extended waiting may allow time for symptom fluctuation, deterioration in quality of life, increased pain, or emergency events, all of which may alter the perceived necessity of surgery (18, 19, 24). However, these explanations should be interpreted cautiously. Because this study did not directly collect patient-reported reasons for no-show, the proposed mechanisms require confirmation in future studies. No-show behavior is likely the result of multiple interacting factors, and future research should incorporate patient-reported reasons to better clarify the pathways through which waiting time may influence attendance behavior.

Existing evidence indicates that optimizing preoperative processes, implementing structured prioritization systems, enhancing communication, and applying personalized reminder strategies can reduce no-show risk (11, 25–28). Additionally, with the development of information technology, machine learning has been applied to predict no-show risk in outpatient endoscopic surgery patients, providing technical support for prospective intervention (29). These findings suggest that earlier identification of patients at higher risk of no-show may be useful, but the effectiveness of specific intervention approaches requires further validation. The operational improvements reported by Quercioli et al. (30), in which dynamic scheduling and resource reallocation reduced day surgery waiting time by approximately 50 percent, demonstrate that systemic interventions can meaningfully shorten waiting periods. In the present study, patients receiving local anesthesia showed greater sensitivity to prolonged waiting time, which may suggest that procedures with lower complexity are more sensitive to delay. This finding indicates that patients with prolonged waiting times may benefit from closer follow-up. The observed increase in risk around the six-day threshold may also provide a useful reference point for designing future intervention studies. Finally, waiting time management must balance efficiency and equity (8). The reduction in waiting time and no-show events achieved through lean management strategies in previous research highlights the potential of systematic quality improvement to enhance access and reliability of care (31).

### Limitations

This study provides new insights, but several limitations should be considered. First, the study was conducted in a single tertiary hospital, which may limit the generalizability of the findings. Second, although we adjusted for a range of demographic, socioeconomic, and clinical variables, residual confounding and bias may still remain because of the retrospective design and the absence of some potentially relevant factors, such as psychological status, prior no-show history, transportation barriers, and informal cancellations not captured in the system. Third, this study lacked direct data on patients’ reasons for no-show, because the retrospective database was primarily designed for clinical documentation and hospital management rather than systematic capture of patient-reported nonattendance reasons. This limitation restricted a deeper understanding of the specific mechanisms through which preoperative waiting time may influence no-show behavior and reduced the empirical basis for targeted intervention strategies. Fourth, sensitivity analyses were not performed to test the robustness of the findings. Fifth, because the primary aim of this study was to evaluate associations rather than to develop a clinical prediction model, formal assessment of model performance, such as discrimination and calibration, was not conducted. Future studies should incorporate sensitivity analyses, assess model performance, and collect patient-reported reasons through questionnaires, interviews, or prospective follow-up to better clarify the mechanisms underlying the observed association between waiting time and no-show risk.

## Conclusion

Based on a large real-world dataset, this study identified a significant nonlinear association between preoperative waiting time and no-show risk among day-surgery patients. In this study population, the association between longer preoperative waiting time and increased no-show risk became more apparent when the waiting time exceeded approximately 6 days. Subgroup analyses suggested possible heterogeneity across patient groups, with middle-aged patients and those receiving local anesthesia showing a stronger association between prolonged waiting time and no-show risk. These findings provide preliminary evidence for understanding the relationship between preoperative waiting time and no-show risk in day surgery. However, given the observational design of this study and the possibility of residual confounding, the findings should be interpreted with caution. Larger prospective studies with more rigorous control of potential confounding factors are needed to further validate these results.

## Data Availability

The raw data supporting the conclusions of this article will be made available by the authors, without undue reservation.
